# Routine blood parameters are helpful for early identification of influenza infection in children

**DOI:** 10.1186/s12879-020-05584-5

**Published:** 2020-11-19

**Authors:** Ronghe Zhu, Cuie Chen, Qiu Wang, Xixi Zhang, Chaosheng Lu, Yuanyuan Sun

**Affiliations:** 1 Department of Pediatrics, the First Affiliated Hospital of Wenzhou Medical University, South Baixiang, Ouhai District, Wenzhou, 325000 Zhejiang China; 2 Department of Pediatrics, Yiwu Maternity and Children Hospital, No. C100 Xinke Road, Yiwu, Jinhua, 322000 Zhejiang China; 3 Department of Pediatrics, People’s Hospital of Yuhuan, No. 18 Changle Road, Yucheng Street, Yuhuan, Taizhou, 317600 Zhejiang China

**Keywords:** Influenza, Routine blood tests, Children, Lymphocyte-to-monocyte ratio

## Abstract

**Background:**

Routine blood parameters, such as the lymphocyte (LYM) count, platelet (PLT) count, lymphocyte-to-monocyte ratio (LMR), neutrophil-to-lymphocyte ratio (NLR), lymphocytes multiplied by platelets (LYM*PLT) and mean platelet volume-to-platelet ratio (MPV/PLT), are widely used to predict the prognosis of infectious diseases. We aimed to explore the value of these parameters in the early identification of influenza virus infection in children.

**Methods:**

We conducted a single-center, retrospective, observational study of fever with influenza-like symptoms in pediatric outpatients from different age groups and evaluated the predictive value of various routine blood parameters measured within 48 h of the onset of fever for influenza virus infection.

**Results:**

The LYM count, PLT count, LMR and LYM*PLT were lower, and the NLR and MPV/PLT were higher in children with an influenza infection (PCR-confirmed and symptomatic). The LYM count, LMR and LYM*PLT in the influenza infection group were lower in the 1- to 6-year-old subgroup, and the LMR and LYM*PLT in the influenza infection group were lower in the > 6-year-old subgroup. In the 1- to 6-year-old subgroup, the cutoff value of the LMR for predicting influenza A virus infection was 3.75, the sensitivity was 81.87%, the specificity was 84.31%, and the area under the curve (AUC) was 0.886; the cutoff value of the LMR for predicting influenza B virus infection was 3.71, the sensitivity was 73.58%, the specificity was 84.31%, and the AUC was 0.843. In the > 6-year-old subgroup, the cutoff value of the LMR for predicting influenza A virus infection was 3.05, the sensitivity was 89.27%, the specificity was 89.61%, and the AUC was 0.949; the cutoff value of the LMR for predicting influenza B virus infection was 2.88, the sensitivity was 83.19%, the specificity was 92.21%, and the AUC was 0.924.

**Conclusions:**

Routine blood tests are simple, inexpensive and easy to perform, and they are useful for the early identification of influenza virus infection in children. The LMR had the strongest predictive value for influenza virus infection in children older than 1 year, particularly in children older than 6 years with influenza A virus infection.

## Background

Influenza is an acute respiratory infectious disease caused by influenza viruses. One billion patients are diagnosed with seasonal influenza each year worldwide, among whom 3 to 5 million have severe cases and as many as 250,000 to 500,000 die [1]. Although most children recover spontaneously from infection, morbidity and mortality rates are higher in children with underlying diseases who are younger than 5 years, particularly children younger than 2 years [2]. However, previously healthy children are also at risk. In the USA, the admission rate of non-high-risk children for influenza was estimated to be 9 per 10,000 children younger than 5 years [3]. According to the WHO, the annual infection rate of children was as high as approximately 50% in the past 11 influenza epidemic seasons [1]. Complications such as pneumonia, myocarditis, septic shock and multiple organ dysfunction, are the main causes of death in children with influenza [4]. Early (within 48 h after infection) use of anti-influenza drugs significantly relieves symptoms, shortens the disease course, and reduces complications. Therefore, the early and rapid diagnosis of influenza and the early use of anti-influenza drugs are essential to improve the prognosis of influenza in children.

The diagnosis of influenza depends on the detection of influenza virus nucleic acids in the respiratory tract, the isolation of influenza virus or the detection of a level of serum-specific antibodies that is at least 4 times the normal level. Common detection methods include virus isolation and culture, RT-PCR and serological detection, all of which have advantages and disadvantages. Virus isolation and culture, and serological detection are time-consuming and not suitable for outpatient screening. RT-PCR takes slightly less time, but is relatively expensive. Viral antigen detection, such as rapid influenza detection, is rapid, simple and displays good specificity; however, the sensitivity is low and it is prone to false negative results [5].

Routine blood tests are the first choice in pediatric fever clinics. They are easy to perform and inexpensive. In recent years, the lymphocyte-to-monocyte ratio (LMR), neutrophil-to-lymphocyte ratio (NLR), mean platelet volume-to-platelet ratio (MPV/PLT) and lymphocytes multiplied by platelets (LYM*PLT) value have been used as new inflammatory markers to predict the prognosis of infectious diseases [6–8], tumors [9, 10] and cardiovascular diseases [11, 12]; these markers have been widely studied in clinical practice.

Some studies have investigated routine blood index values and influenza A infection in adults, but few studies have been conducted in children. In this study, we performed early (within 48 h of the onset of fever) measurements of routine blood parameters in children with fever and influenza-like symptoms, and further explored the predictive value of the LMR, NLR, LYM*PLT, MPV/PLT, lymphocyte (LYM) count and platelet (PLT) count for influenza infection in children.

## Methods

### Patients

Children with fever and influenza-like symptoms aged 0.2 to 14 years who presented at the First Affiliated Hospital of Wenzhou Medical University from January 2018 to February 2020 were included in this study. All the patients underwent routine blood tests and the detection of influenza virus nucleic acids using RT-PCR within 48 h of the onset of fever. Influenza-like symptoms were defined as follows: fever (temperature ≥ 38 °C), cough or sore throat [13]. The exclusion criteria were as follows: (1) systemic chronic diseases, such as diseases of the blood, heart, lung, liver and kidney; (2) immunodeficiency due to a tumor, HIV infection, or the long-term use of oral hormones or immunosuppressive agents; (3) severe or critical illness, namely, the failure of one or more organs; (4) bacterial infections, such as sepsis and suppurative tonsillitis; and (5) Epstein-Barr virus (EBV) infection. This study was approved by the Ethics Committee for Clinical Research of the First Affiliated Hospital of Wenzhou Medical University (No. 2020–065), and all the parents signed the informed consent form.

### Detection of routine blood parameters

Finger prick blood samples were subjected to routine blood tests. A routine analyzer (XN-350, SYSMEX, Hyogo, Kobe, Japan) was used for detection. The LYM count, monocyte (MON) count, PLT count and mean platelet volume (MPV) were recorded. Additionally, other hematological parameters were calculated: the LMR is the ratio of lymphocytes to monocytes, the NLR is the ratio of neutrophils to lymphocytes, the MPV/PLT is the MPV divided by the PLT count, and the LYM*PLT is the lymphocyte count multiplied by the platelet count.

### Detection of influenza virus nucleic acids using RT-PCR

A fully automatic nucleic acid extractor and the associated reagents (Shanghai ZJ Bio-Tech Co., Ltd.) were used to extract all nucleic acids from pharyngeal swabs. Throat swab specimens obtained for the purpose of the influenza virus nucleic acid determination were subjected to RT-PCR, and influenza A and B virus nucleic acid detection kits were used (Z-RR-0097-02, Shanghai ZJ Bio-Tech Co., Ltd.). The amplification system used a final volume of 25 μl, consisting of 19 μl of a mixture of influenza A and B virus nucleic acid fluorescent probes, 1 μl of the enzyme, and 5 μl of the sample. The amplification conditions were as follows: reverse transcription at 45 °C for 10 min, predenaturation at 95 °C for 15 min, denaturation at 95 °C for 15 s, and annealing, elongation and fluorescence detection at 60 °C for 60 s for 45 cycles. All amplification reactions were performed with an ABI7500 quantitative PCR instrument (Applied Biosystems, Inc., USA).

### Statistical analysis

SPSS 25.0 software (SPSS Inc., Chicago, IL, USA) was used to analyze the data. Continuous variables were expressed as the mean ± standard when normally distributed or the median (interquartile range) when non-normally distributed. Categorical variables were expressed as frequencies. Comparisons of continuous variables groups were performed with t-tests or Wilcoxon tests. The One-way ANOVA or Kruskal-Wallis test was used to analyze differences in continuous variables among groups. The Pearson χ2 test or Fisher’s exact test was applied to analyze categorical variables. A receiver operating characteristic (ROC) curve was constructed to evaluate the diagnostic value of the LYM count, PLT count, LMR, NLR, LYM*PLT and MPV/PLT for influenza A and B virus infection. Statistical significance was indicated by *P* < 0.05.

## Results

### Patient characteristics

The diagnosis of influenza infection is based on the presence of influenza-like symptoms with a positive RT-PCR result for influenza A or B [14]. In the present study, 388 children with influenza A virus infection (A+ group), 169 children with influenza B virus infection (B+ group), 198 children with influenza-like symptoms who were negative for both influenza A and B viruses (A-B- group) and 259 healthy children (H group) who underwent physical examinations at the same time were included. All children were divided into three groups according to age: the < 1-year-old group, the 1- to 6-year-old group and the > 6-year-old group. In each age group, statistically significant differences in the age and sex distributions were not observed among the four clinical groups (A+, B+, A−/B- and H) (*P* > 0.05), only 3 patients were included in the < 1-year-old B+ group, and thus a statistical analysis was not performed (Table 1).

**Table 1:** The baseline characteristics of patients

		A+ group (*n* = 388)	B+ group (*n* = 169)	A- /B- group (*n* = 198)	H group (*n* = 259)	χ2/H	*P*
< 1 year old	Males	11	1	12	21	0.384	0.825
	Females	7	2	7	17		
	Mean age (y)	0.8 (0.5–0.8)	0.6 ± 0.2	0.7 (0.6–0.9)	0.6 (0.5–0.8)	2.146	0.342
1–6 years old	Males	98	24	49	62	1.218	0.749
	Females	95	29	53	54		
	Mean age (y)	3.7 (2.4–4.8)	4.0 (3.1–4.9)	3.4 (2.4–4.6)	3.4 (2.4–5.1)	3.740	0.291
> 6 years old	Males	104	53	33	57	2.415	0.060
	Females	73	60	44	48		
	Mean age (y)	8.6 (7.2–11.8)	7.9 (7.1–9.8)	8.6 (6.8–11.7)	9.4 (7.5–11.5)	6.531	0.088

A+ group, influenza A virus infection; B+ group, influenza B virus infection; A-B- group, influenza-like symptoms who were negative for both influenza A and B viruses; H group, healthy children

### Differences in routine blood parameters among the clinical groups in the three age groups

**Fig. 1:**
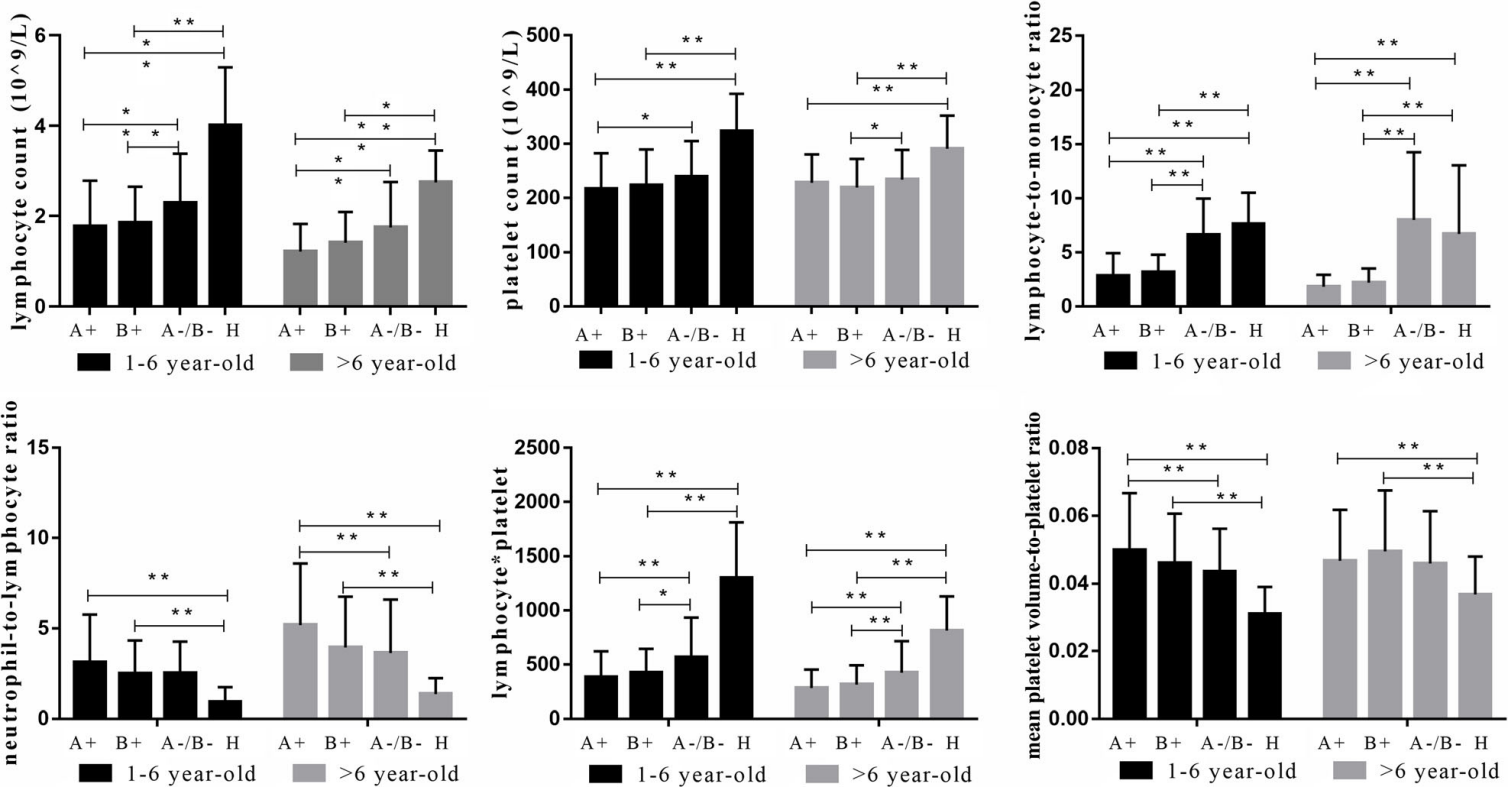
Differences in lymphocyte (LYM) count, platelet (PLT) count, lymphocyte-to-monocyte ratio (LMR), neutrophil-to-lymphocyte ratio (NLR), lymphocyte*platelet (LYM*PLT) and mean platelet volume-to-platelet ratio (MPV/PLT) values in the A+, B+, A-B- and H group. * *P* < 0.05, ** *P* < 0.01

Only 3 patients were included in the < 1-year-old B+ group, and thus a statistical analysis was not performed (Table 2). The red blood cell count (RBC) and hemoglobin (Hb) level in the A+ group, B+ group, A-B- group and H group were not significantly different in the subgroups of patients < 1 year old, 1–6 years old and > 6 years old (Tables 2, 3 and 4). Compared with the H group, the A+ group, B+ group, and A-B- group had lower LYM counts, PLT counts, LMRs and LYM*PLT values, and higher NLRs and MPV/PLT values (Tables 2, 3 and 4). In the < 1-year-old subgroup, no significant differences in the LYM count, PLT count, LMR, NLR, LYM*PLT and MPV/PLT were observed between the A+ group and A-B- group. In the 1- to 6-year-old subgroup, the LYM count, PLT count, LMR, LYM*PLT and MPV/PLT were significantly different between the A+ group and the A-B- group, and the LYM count, LMR and LYM*PLT were significantly different between the B+ group and the A-B- group (Fig. 1 and Table 3). In the > 6-year-old subgroup, the LYM count, LMR, NLR and LYM*PLT were significantly different between the A+ group and the A-B- group; the PLT count, LMR and LYM*PLT were significantly different between the B+ group and the A-B- group (Fig. 1 and Table 4).

**Table 2:** Hematological parameters of the four groups in the < 1-year-old group

Parameters	A+ group (1)	B+ group	A−/B- group (2)	H group (3)	F/H	*P*
WBC (10^9^/L)	7.25 ± 2.62^a^	7.62 ± 2.81	6.49 ± 1.89	9.14 ± 2.07	10.939	0.000
NEU (%)	45.26 ± 13.36^a^	34.00 ± 16.70	37.53 ± 16.13	19.74 ± 8.22	39.214	0.000
LYM (%)	36.72 ± 12.79^a^	51.00 ± 19.08	45.58 ± 17.76	68.05 ± 8.34	42.247	0.000
LYM(10^9^/L)	2.72 ± 1.49^a^	3.98 ± 2.63	2.88 ± 1.36	6.28 ± 1.84	41.597	0.000
MON (%)	16.44 ± 4.88^a^	14.00 ± 2.00	14.21 ± 3.91	7.18 ± 2.68	48.749	0.000
MON (10^9^/L)	1.18 ± 0.53^a^	1.02 ± 0.26	0.89 ± 0.29	0.65 ± 0.25	21.414	0.000
NLR	1.57 ± 1.18^a^	0.84 ± 0.74	1.14 ± 1.03	0.31 ± 0.18	40.717	0.000
LMR	2.09 (1.50–3.02)^a^	3.80 ± 1.86	2.55 (1.94–3.49)	10.28 (8.15–11.62)	52.816	0.000
RBC (10^12^/L)	4.50 ± 0.51	4.56 ± 0.26	4.56 ± 0.32	4.44 ± 0.80	0.120	0.887
Hb (g/dl)	12.05 ± 0.10	11.93 ± 0.64	12.10 ± 0.73	12.03 ± 0.74	0.260	0.878
PLT (10^9^/L)	284.56 ± 66.36^a^	287.33 ± 85.85	264.47 ± 60.26	366.39 ± 91.08	13.117	0.000
LYM*PLT	781.16 ± 508.13^a^	1040.84 ± 461.46	741.64 ± 339.72	2379.39 ± 1134.19	45.140	0.000
MPV/PLT	0.037 ± 0.013	0.036 ± 0.014	0.038 ± 0.009	0.028 ± 0.008	9.469	0.000

*WBC* White blood cell count, *NEU* NeutrophilL, *LYM* Lymphocyte, *MON* Monocyte, *NLR* Neutrophil-to-lymphocyte, *LMR* Lymphocyte-to-monocyte, *RBC* Red blood cell count, *Hb* Hemoglobin, *PLT* Platelet, LYM*PLT Lymphocyte* platelet, *MPV/PLT* Mean platelet volume/platelet ratio

^a^Compared with the H group, *P* < 0.05

**Table 3:** Hematological parameters of the four groups in the 1- to 6-year-old group

Parameters	A+ group	B+ group	A−/B- group	H group	F/H	*P*
WBC (10^9^/L)	6.55 ± 2.33^ab^	6.35 ± 2.50^ab^	7.77 ± 2.94	7.94 ± 1.77	44.353	0.000
NEU (%)	59.44 ± 15.72^a^	56.69 ± 14.53^a^	57.69 ± 14.60	38.33 ± 11.82	124.595	0.000
LYM (%) 0.048	28.43 ± 14.16^a^	30.94 ± 12.60^a^	31.13 ± 13.35	50.85 ± 12.38	74.420	0.000
LYM (10^9^/L)	1.53 (1.05–2.11)^ab^	1.68 (1.28–2.37)^ab^	2.13 (1.44–3.00)	3.89 (3.04–4.99)	185.768	0.000
MON (%)	11.00 (8.00–14.00)^a^	11.00 (7.65–14.15)^a^	9.00 (8.00–12.00)	7.00 (6.00–8.00)	96.138	0.000
MON (10^9^/L)	0.62 (0.47–0.89)^a^	0.60 (0.44–0.84)^a^	0.69 (0.50–0.94)	0.55 (0.44–0.65)	22.076	0.000
NLR	2.38 (1.33–3.95)^a^	1.86 (1.14–3.08)^a^	2.01 (1.1–3.71)	0.74 (0.49–1.11)	134.530	0.000
LMR	2.47 (1.58–3.38)^ab^	2.93 (1.95–4.07)^ab^	5.88 (4.01–8.71)	6.96 (5.85–9.09)	235.215	0.000
RBC (10^12^/L)	4.58 ± 0.34	4.59 ± 0.33	4.56 ± 0.31	4.62 ± 0.28	0.731	0.534
Hb (g/dl)	12.59 ± 0.81	12.59 ± 0.81	12.47 ± 0.73	12.63 ± 0.67	1.031	0.379
PLT (10^9^/L)	216.99 ± 65.96)^ab^	225.57 ± 64.79)^a^	239.40 ± 65.54	322.91 ± 69.79	65.139	0.000
LYM*PLT	316.14 (209.46–496.97)^ab^	383.61 (229.00–566.41)^ab^	446.81 (305.83–730.05)	1210.24 (957.75–1655.55)	217.522	0.000
MPV/PLT	0.046 (0.039–0.058)^ab^	0.045 (0.034–0.055)^a^	0.043 (0.035–0.049)	0.030 (0.025–0.035)	132.718	0.000

^a^Compared with the H group, *P* < 0.05

^b^Compared with the A−/B- group, *P* < 0.05

**Table 4:** Hematological parameters of the four groups in the > 6-year-old group

Parameters	A+ group	B+ group	A−/B- group	H group	F/ H	*P*
WBC (10^9^/L)	6.86 ± 2.11	6.70 ± 2.20	7.44 ± 2.68	6.90 ± 1.75	4.400	0.221
NEU (%)	69.16 ± 11.95^ab^	65.21 ± 12.56^a^	62.46 ± 15.42	48.37 ± 10.03	141.857	0.000
LYM (%)	16.20 (11.25–24.85)^ab^	20.20 (14.20–27.30)^a^	23.30 (16.00–31.55)	41.30 (35.60–47.65)	175.453	0.000
LYM(10^9^/L)	1.22 ± 0.61^ab^	1.41 ± 0.67^a^	1.74 ± 1.01	2.74 ± 0.71	187.333	0.000
MON(%)	10.97 ± 3.80^a^	11.23 ± 4.23^a^	10.35 ± 4.26	7.09 ± 2.21	90.097	0.000
MON(10^9^/L)	0.74 ± 0.32^a^	0.73 ± 0.31^a^	0.70 ± 0.26	0.48 ± 0.16	69.427	0.000
NLR	4.41 (2.53–6.83)^ab^	3.35 (2.11–5.17)^a^	2.63 (1.73–4.68)	1.16 (0.87–1.56)	165.854	0.000
LMR	1.59 (1.06–2.26)^ab^	1.94 (1.34–2.61)^ab^	6.15 (4.11–9.62)	5.78 (4.47–7.38)	281.453	0.000
RBC (10^12^/L)	4.72 ± 0.49	4.76 ± 0.55	4.69 ± 0.31	4.78 ± 0.36	1.957	0.120
Hb (g/dl)	13.29 ± 0.88	13.37 ± 0.88	13.26 ± 0.88	13.44 ± 0.95	0.819	0.484
PLT (10^9^/L)	227.70 ± 52.55^a^	218.50 ± 53.60^ab^	235.36 ± 56.33	291.42 ± 61.12	38.839	0.000
LYM*PLT	242.16 (157.50–367.07)^ab^	265.86 (188.80–398.38)^ab^	337.69 (242.28–547.83)	769.29 (576.34–998.95)	196.469	0.000
MPV/PLT	0.044 (0.037–0.055)^a^	0.045 (0.038–0.056)^a^	0.042 (0.035–0.054)	0.034 (0.031–0.040)	59.784	0.000

^a^Compared with the H group, *P* < 0.05

^b^Compared with the A−/B- group, *P* < 0.05

### Predictive value of the LYM count, PLT count, LMR, NLR, LYM*PLT, and MPV/PLT influenza infection

#### The 1- to 6-year-old a+ group

**Fig. 2:**
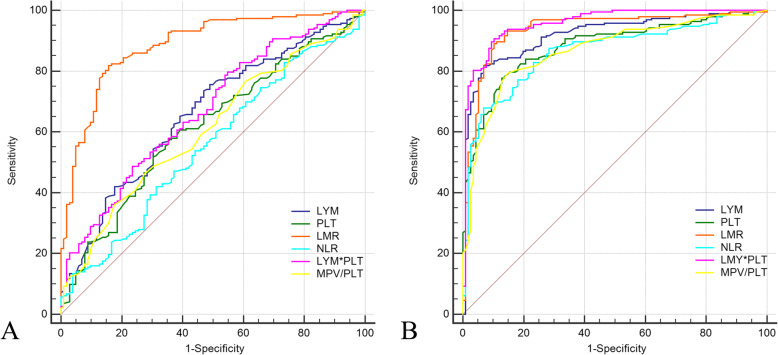
ROC curves of LYM count, PLT count, LMR, NLR, LYM*PLT and MPV/PLT values in the 1- to 6 -year -old A+ group (**a**. the A-B- group as a reference; **b**. the H group as the reference)

The variable that best predicted positivity for influenza virus A infection based on the area under the curve (AUC) was the LMR. When the A-B- group was used as a reference, the cutoff value was 3.75, the AUC was 0.886, and the sensitivity and specificity were 81.87 and 84.31%, respectively. When the H group was used as the reference, the maximum AUC was obtained for the LYM*PLT (followed by the LMR); the cutoff value of the LYM*PLT was 680.48, the AUC was 0.958, and the sensitivity and specificity were 90.67 and 89.66%, respectively (Fig. 2).

#### The 1- to 6-year-old B+ group

**Fig. 3:**
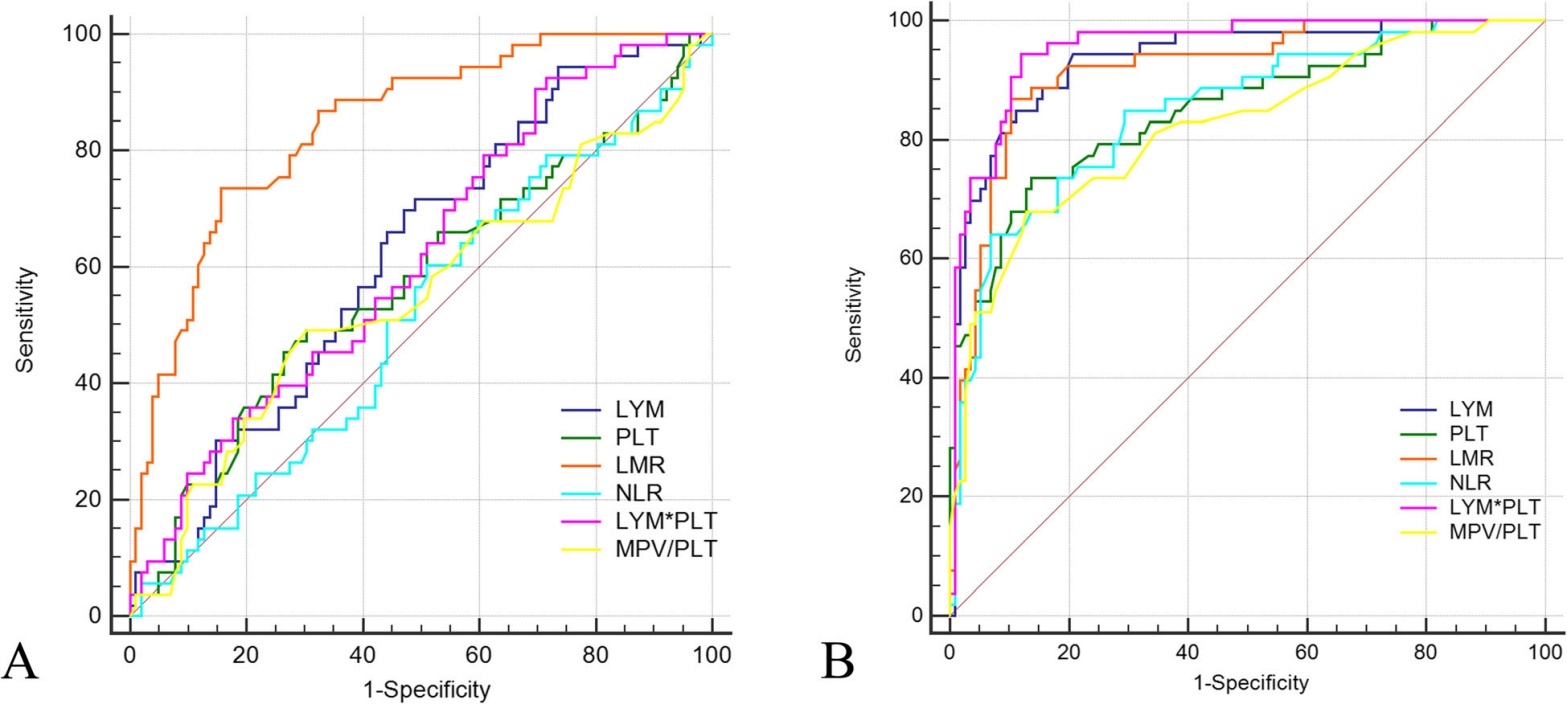
ROC curves of LYM count, PLT count, LMR, NLR, LYM*PLT and MPV/PLT values in the 1- to 6 -year -old B+ group (**a**. the A-B- group as a reference; **b**. the H group as the reference)

The variable that best predicted positivity for influenza virus B based on the AUC was the LMR. When the A-B- group was used as a reference, the cutoff value was 3.71, the AUC was 0.843, and the sensitivity and specificity were 73.58 and 84.31%, respectively. When the H group was used as the reference, the cutoff value for the LMR was 4.47, the AUC was 0.918, and the sensitivity and specificity were 86.79 and 89.66%, respectively (Fig. 3).

#### The > 6-year-old a+ group

**Fig. 4:**
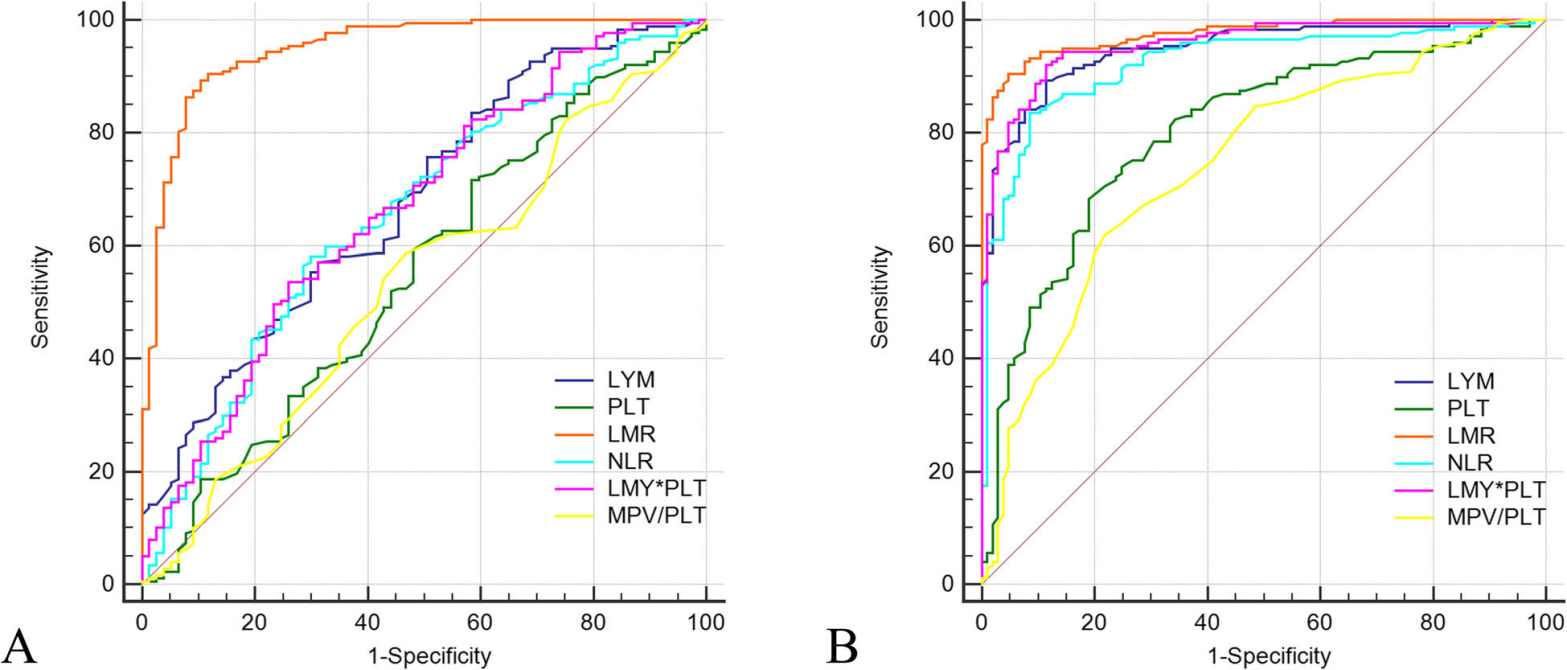
ROC curves of LYM count, PLT count, LMR, NLR, LYM*PLT and MPV/PLT values in the > 6 -year -old A+ group (**a**. the A-B- group as a reference; **b**. the H group as the reference)

The maximum AUC was obtained for the LMR. Using the A-B- group as a reference, the cutoff value was 3.05, the AUC was 0.949, and the sensitivity and specificity were 89.27 and 89.61%, respectively. When the H group was used a reference, the cutoff value was 3.09, the AUC was 0.975, and the sensitivity and specificity were 90.40 and 95.24%, respectively (Fig. 4).

#### The > 6-year-old B+ group

**Fig. 5:**
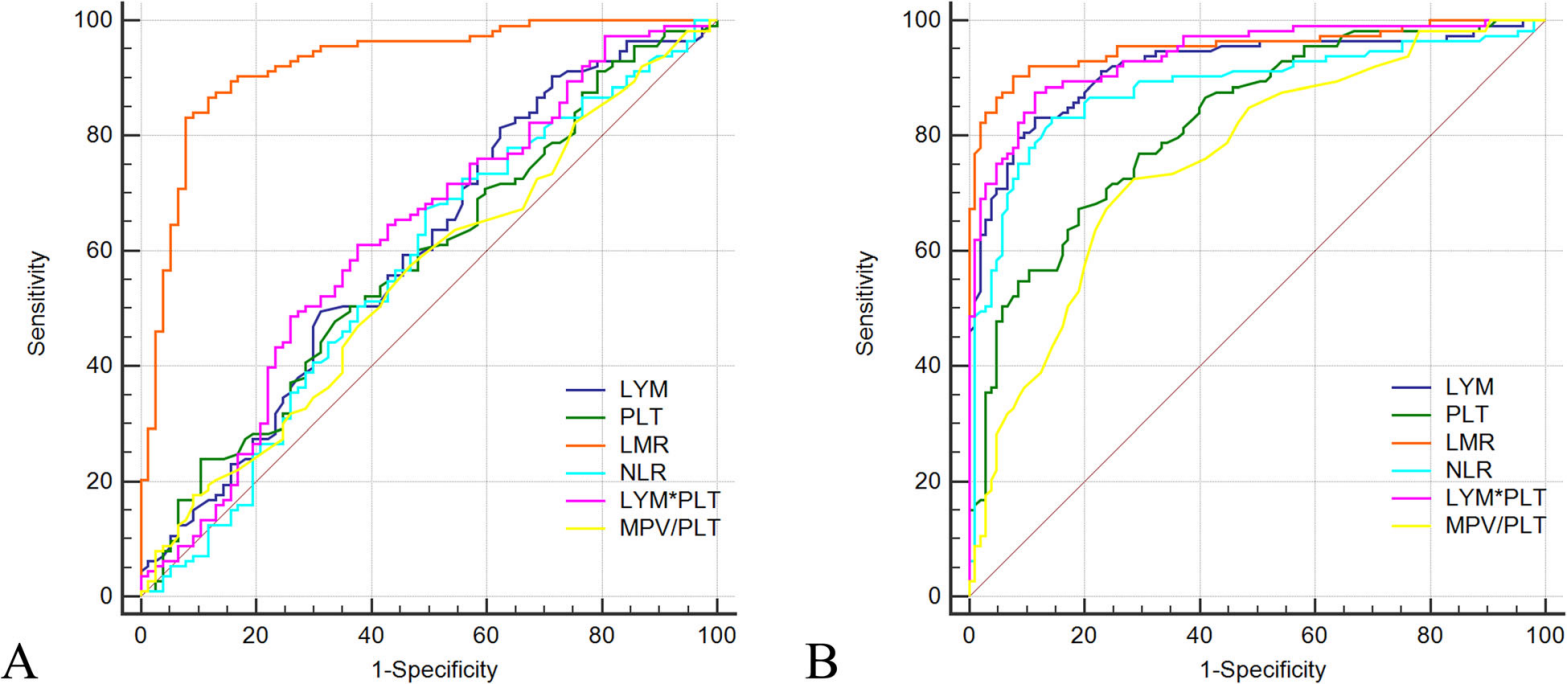
ROC curves of LYM count, PLT count, LMR, NLR, LYM*PLT and MPV/PLT values in the > 6 -year -old B+ group (**a**. the A-B- group as a reference; **b**. the H group as the reference)

The maximum AUC was obtained for the LMR. Using the A-B- group as a reference, the cutoff value was 2.88, the AUC was 0.924, and the sensitivity and specificity were 83.19 and 92.21%, respectively. When the H group was used a reference, the cutoff value of the LMR was 3.48, the AUC was 0.954, and the sensitivity and specificity were 90.27 and 92.38%, respectively (Fig. 5).

## Discussion

Influenza viruses can be divided into three types according to the nucleocapsid protein and the matrix protein antigen: A, B and C. No cross-immunity exists among these types. The type with the greatest antigen variability is influenza A, which often causes regional outbreaks and epidemics and might even cause a global pandemic. Over the past 100 years, influenza A outbreaks have occurred seasonally and have caused several global pandemics. The most serious pandemic was the Spanish H1N1 influenza pandemic in 1918, which killed 50 million people [15, 16]. Influenza B has weak antigen variability, and it often causes moderate epidemics or local outbreaks. Influenza C rarely infects humans [17]. The clinical symptoms of influenza virus infections in children are similar to infections with other respiratory pathogens and are nonspecific, including a high fever, chills, muscle aches, sore throat, cough, and runny nose. Gastrointestinal symptoms such as vomiting, abdominal pain, and diarrhea are relatively common in children infected with influenza B [14]. Patients with mild symptoms typically recover within a short time, and patients with severe symptoms rapidly develop dyspnea accompanied by refractory hypoxemia and may eventually develop acute respiratory distress syndrome, septic shock, heart failure, acute necrotizing encephalopathy, and multiple organ dysfunction, which are life-threatening and even fatal conditions [18]. Therefore, a rapid and simple index for the early diagnosis of influenza infection in children is needed.

The diagnosis of influenza mainly depends on the detection of viral nucleic acids and antibodies. Virus isolation and culture were previously the “gold standard” for the diagnosis of influenza, but this process is time-consuming and expensive, has high technical requirements and is difficult to perform. RT-PCR is the most effective nucleic acid detection technology for influenza, with a sensitivity and specificity as high as 98.5 and 100% [19], respectively. RT-PCR has now become the “gold standard” for the diagnosis of influenza. Although RT-PCR takes less time than virus isolation and culture, it is still expensive and takes several hours, limiting its use as a routine method for screening for influenza in pediatric fever clinics. Serological testing requires two serum samples from both the acute phase and the convalescent phase. Convalescent blood samples should be collected 2–4 weeks after the onset of the disease. If the antibody level is more than 4 times higher in the convalescent phase than in the acute phase, the patient is diagnosed with influenza. Obviously, this approach is not suitable for influenza screening in outpatients. Influenza virus antigen detection, such as rapid influenza diagnostic tests, are able to be performed within 30 min, but the sensitivity is only 40–70% [5]. Routine blood tests are the most common tests performed in pediatric fever clinics and have been used as the primary method for identifying bacterial and viral infections. In recent years, researchers have performed an in-depth analysis of various routine blood parameters, and these parameters are useful for the early diagnosis and prognostic assessment of other diseases [6–12].

Influenza strains infect respiratory epithelial cells, and the attachment of the virus to cells via sialic acid receptors enables uptake of the virus into the cells, followed by the recognition of the virus by pattern recognition receptors (PRRs). PRRs trigger cytokine responses and protective immunity, but they might also contribute to immune pathology [20]. Lymphocytes are the main immune cells involved in the elimination of viruses. In conventional viral infections, the proportion of lymphocytes in the circulation is usually increased. Previous studies have reported a significant decrease in LYM counts in patients infected with influenza A [8, 21, 22], but few studies have examined LYM counts in patients infected with influenza B. According to Nichols et al. [23], LYM induced self-apoptosis by regulating the expression of FasL on the cell surface and released soluble FasL after influenza infection, leading to a decrease in the LYM count. In the present study, a significantly lower LYM count was observed in children with an influenza A or B infection than in children aged 1–6 years with influenza-like symptoms who tested negative for influenza A and B viruses, and no significant difference in the LYM count was observed between children with influenza A and influenza B infections. In children > 6 years old infected with influenza A, the LYM count was significantly lower than in children who were not infected with influenza viruses. The LYM count was not significantly different between children infected with influenza virus B and children who were not infected with either influenza A or B viruses. Lewis et al. [24] suggested that lymphopenia is mainly due to a reduction in T cells and, to a lesser extent, B cells and has a short duration.

Leukocytes such as neutrophils and monocytes (MON) protect the host from influenza infection by releasing preformed cytokines, and the granule contents help hosts eliminate the threat posed by replicating viruses. Coskun O et al. [25] recommended monocytosis as a surrogate marker for infection with influenza A virus. In the present study, there was no statistically significant difference in the MON count between children older than 1 year who were infected with either influenza A or B viruses and children who were not infected with influenza viruses. The LMR is the ratio of the LYM count to the MON count, and an LMR < 2 has been used as a surrogate marker for influenza A infection [26, 27]. Based on the findings from the present study, the LMR is the best index for the prediction of influenza virus infection. At the same time, the AUCs for the prediction of influenza A and influenza B infections were higher in children aged > 6 years than in children aged 1–6 years, suggesting that the diagnostic value of the LMR for influenza is greater in children aged > 6 years than in children aged < 6 years. In addition, the AUC of the LMR for predicting an influenza A infection was higher than that for predicting an influenza B infection in the > 1-year-old subgroup, indicating that the predictive value of the LMR was greater for influenza A than that for influenza B.

PLT have been reported regulate host immunity and complement responses in the initial intrinsic defense against influenza virus infection [28], and researchers have explored the different mechanisms by which virus infection can interfere with PLT production and might trigger PLT destruction [29], subsequently decreasing the PLT count. In the present study, a significantly lower PLT count was observed in the influenza A group than in the group of children who were negative for both influenza A and influenza B in the 1- to 6-year-old subgroup, consistent with the results reported by Fei et al. [8]. However, there was no significant difference between the group infected with influenza B and the group of children who were negative for both influenza A and influenza B. Among the children older than 6 years, the PLT count was not statistically significantly different between the group infected with influenza A, the group infected with influenza B and the group of children who were negative for both influenza A and influenza B. Thus, the PLT count has predictive value only for children aged 1 to 6 years who are infected with influenza A; furthermore, its predictive value is low, even in that group, with an AUC of 0.615 and sensitivity and specificity of 58.03 and 63.73%, respectively. In recent years, researchers have attempted to combine the PLT count with other indicators, such as LYM*PLT and MPV/PLT, for disease prediction [30]. Fei et al. [8] reported better predictive value of the LYM*PLT and MPV/PLT for children aged < 6 years who were infected with influenza A, and the predictive value was greater than the LMR. However, the LMR had the highest predictive value for influenza infection, followed by the LYM*PLT, PLT count, and MPV/PLT, among children over the age of 1 year.

Recently, the NLR was shown to be positively associated with systemic inflammation [6], acute pancreatitis [31], liver disease [32] and rheumatic diseases [33]. The NLR exhibits a high sensitivity for the detection of influenza virus infection [34]. In the present study, a significant difference in the NLR was not observed among the group infected with influenza A, the group infected with influenza B and the group of children who were negative for both influenza A and influenza B in the 1–6-year-old subgroup. In the subgroup of children aged > 6 years, the NLR was significantly higher in the group infected with influenza A than in the group negative for both influenza A and influenza B. However, no significant difference was observed between the group infected with influenza B and the group of children who were negative for both influenza A and influenza B in the > 6-year-old subgroup. Based on our findings, the NLR has predictive value only for children aged > 6 years who are infected with influenza A, with an AUC of 0.657 and a sensitivity and specificity of 58.19 and 70.13%, respectively. In addition, no significant differences in the LYM count, PLT count, LMR, NLR, LYM*PLT and MPV/PLT were observed between the group infected with influenza A and the group of children who were negative for influenza A and influenza B in the < 1-year-old subgroup. Due to the small sample size, a statistical analysis was not performed to compare the influenza B group with the other groups among children < 1 year old. Thus, the routine blood parameters displayed poor predictive value for influenza in the < 1-year-old age group. We propose that this discrepancy may be related to the immune function and development of blood cells in children, and requires further investigation.

## Conclusions

A significantly lower LMR was observed in children older than 1 year who had influenza, particularly children older than 6 years infected with influenza A, than in children without influenza. The LMR is potentially useful as an early predictor of influenza A infection in children older than 6 years, with an AUC of 0.949, a sensitivity of 89.27% and a specificity of 89.61%.

## Data Availability

All data generated or analysed during this study are included in this published article.

## Abbreviations

LYM: Lymphocyte
PLT: Platelet
LMR: Lymphocyte-to-monocyte ratio
NLR: Neutrophil-to-lymphocyte ratio
MPV/PLT: Mean platelet volume-to-platelet ratio
AUC: Area under the curve
LYM*PLT: Lymphocytes multiplied by platelets
EBV: Epstein-Barr virus
ROC: Receiver operating characteristic
A+ group: Influenza A virus infection
B+ group: Influenza B virus infection
A-B- group: Negative for both influenza A and B viruses
H group: Healthy children
RBC: Red blood cell count
Hb: Hemoglobin
PRRs: Pattern recognition receptors
MON: Monocytes
